# Review and Perspectives on *Bifidobacterium lactis* for Infants’ and Children’s Health

**DOI:** 10.3390/microorganisms11102501

**Published:** 2023-10-05

**Authors:** Annie Tremblay, Stéphane Bronner, Sylvie Binda

**Affiliations:** 1 Rosell Institute for Microbiome and Probiotics, 6100 Royalmount Avenue, Montreal, QC H4P 2R2, Canada; atremblay@lallemand.com (A.T.); sbronner@lallemand.com (S.B.); 2 Lallemand Health Solutions, 19 Rue des Briquetiers, BP 59, 31702 Toulouse, France

**Keywords:** probiotics, *Bifidobacteria*, *Bifidobacterium lactis*, pediatrics, early life dysbiosis, infant nutrition

## Abstract

The influence of microbiota dysbiosis in early life is increasingly recognized as a risk factor for the development of several chronic diseases later in life, including an increased risk of asthma, eczema, allergies, obesity, and neurodevelopmental disorders. The question whether the potential lifelong consequences of early life dysbiosis could be mitigated by restoring microbiota composition remains unresolved. However, the current evidence base suggests that protecting the normal development of the microbiome during this critical developmental window could represent a valuable public health strategy to curb the incidence of chronic and lifestyle-related diseases. Probiotic *Bifidobacteria* are likely candidates for this purpose in newborns and infants considering the natural dominance of this genus on microbiota composition in early life. Moreover, the most frequently reported microbiota composition alteration in association with newborn and infant diseases, including necrotizing enterocolitis and diarrhea, is a reduction in *Bifidobacteria* levels. Several studies have assessed the effects of *B. animalis* subsp. *lactis* strains in newborns and infants, but recent expert opinions recommend analyzing their efficacy at the strain-specific level. Hence, using the B94 strain as an example, this review summarizes the clinical evidence available in infants and children in various indications, discussing the safety and potential modes of actions while providing perspectives on the concept of “non-infant-type” probiotics for infants’ health.

## 1. Introduction

The concept that microbiota establishment begins in utero has recently emerged based on the detection of microbial particles in the placenta, amniotic fluid, and meconium, challenging the well-established belief that the womb is a sterile environment [1,2]. While the existence of a maternal–fetal gut microbiota axis is gaining acceptance [2], the mechanisms involved (i.e., maternal transfer of actual bacteria or only their particles/metabolites) and the belief that contamination could be the source of the low biomass found in niches previously considered sterile such as the placenta are still a matter of debate [3,4]. However, the notion that the newborn gut microbiota is mainly acquired from the mother during vaginal birth and breastfeeding remains widely accepted [5,6]. In line with this, factors such as delivery circumstances (i.e., C-section (CS) or younger gestational age at birth), perinatal antibiotic exposure, or nutrition and feeding mode (breast milk, formula, or mixed feeding) are recognized as the main factors influencing microbiota seeding and maturation during the first years of life [5]. Additional factors shown to influence the establishment and maturation of the infant microbiota include the mother’s general and metabolic health and diet, birth or living environment (home or hospital), contact with siblings and furry pets, and ethnicity [7]. The introduction of solid foods (or more precisely, the progressive reduction and cessation of breastfeeding) was also shown to influence the maturation and diversification of an infant’s gut microbiota towards the more stable and adult-like composition seen around 3 years old [8,9]. Despite the complexity and interindividual variability resulting from the many potential combinations of these modulating factors, several studies have shown that development of the microbiome in early life is a non-random, sequential process [10,11,12] which appears to be driven by the ecological concept of priority effects [13,14]. Although more research is required to define the specific determinants of priority effects dictating microbial successions in the first years of life and afterwards [15], a well-known example of such effect is supported by numerous studies; the first colonizers of the neonatal gut usually belong to the Pseudomonadota and Actinomycetota phyla, with a predominance of facultative anaerobes like Bacillota (*Enterococcus*, *Staphylococcus*, *Streptococcus*) and Pseudomonadota (*Enterobacter*, *Escherichia coli*). These species are thought to reduce oxygen levels in the gut to allow subsequent colonization by obligate anaerobes such as Actinomycetota (mainly *Bifidobacteria*), Bacteroidota (*Bacteroides*), and Bacillota (*Clostridium*, *Lactobacillus*, *Ruminococcus*) [4].

Microbiota composition analyses have identified *B. breve*, *B. bifidum*, *B. longum*, *B. adolescentis*, *B. pseudolongum*, *B. pseudocatenulatum*, and *B. animalis* subsp. *lactis* (*B. lactis*) as the most abundant *Bifidobacterium* species in the human colon in an age-dependent manner [16]. While *Bifidobacteria* dominate microbiota composition during early life, they are no longer among the top taxa in adults. In addition, there is an age-dependent switch in the relative abundance of specific *Bifidobacterium* species; typically, *B. longum*, *B. breve*, and *B. bifidum* usually dominate during infancy, while *B. catenulatum*, *B. adolescentis*, and *B. longum* are more abundant during adulthood [17]. Recently, *B. lactis* was detected among the eight most abundant *Bifidobacterium* species in the feces of full-term and premature newborns during the first 3 months of life, albeit at lower levels than *B. longum* and *B. breve* [18]. During the first 2 weeks of life (sampling at 2 and 10 days), the proportion of *B. lactis* among total *Bifidobacteria* was increased significantly in premature versus full-term newborns and tended to be higher at these time points in CS-delivered versus vaginally delivered full-term newborns [18]. While this could suggest an impact of feeding mode in relation with CS or premature birth, there was no significant difference in the relative abundance of *B. lactis* between vaginally delivered, full-term babies who were either exclusively breastfed or received mixed breastmilk/formula feeding at these time points. However, *B. lactis* tended to increase at 3 months of age in the latter group [18]. Other studies also reported a higher relative abundance or prevalence of *B. lactis* in CS-born full-term or premature newborns compared to their respective vaginally delivered counterparts [11,19,20]. Although the detection of *B. lactis* within the human microbiota was suggested to reflect the widespread use/presence of these strains in dairy products and fermented foods rather than a true autochthonous nature, its presence in feces alongside recognized autochthonous infant-type strains in various pediatric populations without the explicit mention of supplementation warrants a better understanding of its modes of action and beneficial effects through in vitro, in vivo, and clinical studies.

This review summarizes the preclinical evidence available on *B. lactis* B94 and describes the clinical evidence about this strain in pediatric populations on necrotizing enterocolitis, diarrhea in acute gastroenteritis, and other GI symptoms in irritable bowel syndrome, as well as on antibiotic-associated diarrhea in *H. pylori* eradication. We also put into perspectives the concept of infant- versus non-infant-type probiotics, such as the documented *B. lactis* strains, for the health of infants and children.

## 2. *Bifidobacterium lactis* B94 and Pediatric Gut Health

As summarized in Table 1, the in vitro and in vivo preclinical studies suggest that *B. lactis* B94 exerts its beneficial effects by protecting the intestinal lining morphological integrity and function, modulating immune responses, and potentially competing with bacterial pathogens. Furthermore, the capacity of *B. lactis* B94 to produce high levels of acetate in vitro and to increase acetate levels in vivo could mediate some of its competitive and beneficial effects, as shown for other *Bifidobacteria* [21]. These mechanisms of action could explain the significant health benefits observed with *B. lactis* B94 in clinical studies conducted in pediatric populations, which include necrotizing enterocolitis, diarrhea in acute gastroenteritis, GI symptoms in IBS, *H. pylori* eradication, and antibiotic-induced diarrhea.

### 2.1. Necrotizing Enterocolitis Incidence and Progression

Considering that inulin was previously shown to support the growth of B94 in vitro and exerts a bifidogenic effect in newborns [36,46], it was combined with B94 in three studies on the incidence of necrotizing enterocolitis (NEC) in populations at higher risk of NEC, namely preterm babies (two studies) and term newborns with cyanotic congenital heart disease (CCHD) (one study). Babies with CCHD are at higher risk of developing NEC with an incidence between 3–9%, which is comparable to the global NEC incidence reported for premature and VLBW infants (2–13%) [47].

The ProPre-Save study was a large multicenter RCT including 400 very-low birth weight (VLBW) neonates from five hospitals [48]. Eligible babies (gestational age < 32 weeks; birth weight < 1500 g) had to be born in the NICU or transferred within the first week of life and have started enteral feeding before inclusion. *B. lactis* B94, either alone (n = 100) or in combination with inulin (n = 100), significantly reduced the incidence of Bell stage ≥ 2 NEC (incidence of 2.0% and 4.0% respectively, *p* < 0.001) compared to the control groups receiving inulin only (incidence of 12.0%; n = 100) or placebo (18.0%, n = 100) [48]. There was no difference in adverse events between groups, supporting an excellent safety profile in this vulnerable population. Infants in the placebo group remained in the NICU significantly longer (*p* = 0.003) and mortality rate was significantly higher (*p* = 0.002) than in the B94-supplemented groups. Out of 20 deaths overall, 15 were attributed to sepsis and multi-organ failure (of which 12 occurred in the placebo group). It is to be noted, however, that the placebo group displayed more risk factors at baseline (e.g., slightly lower birth weight, slightly higher SNAPPE-II scores, longer antibiotic exposure, more infants with patent ductus arteriosus and intraventricular hemorrhage), suggesting that the mortality rate in this group could have been increased compared to the other groups because of a more severe disease condition. Nevertheless, when considering the inulin only group as control (which has identical baseline characteristics to the probiotic groups), the protective effect of B94 on NEC remains significant.

Another study in preterm newborns (<35 weeks GA, <2500 g BW) showed that *B. lactis* B94 may improve weight gain in premature infants, with a daily median weight gain significantly higher in the probiotic group than in the control group (17.2 g/day vs. 14.5 g/day; *p* = 0.038) [49]. Upon stratification by birth weight categories, the faster weight gain was observed in the 1501–2500 g category (low birth weight), rather than in the more severely premature categories <1000 g and 1000–1500 g (extremely low and very low birth weight) for whom the weight gain was similar between groups. In this study, an imbalance in baseline characteristics between groups (i.e., lower baseline birth weight, lower 1 min AGSPAR score, and significantly more infants being small for gestational age in the probiotic group) may have resulted in the higher incidence of sepsis in this group (68.1% vs. 60.5%; *p* = 0.008). However, although Stage 1 NEC incidence was similar between groups (10.6% vs. 14.3%, *p* = 0.42), none of the infants receiving *B. lactis* B94 progressed to Stage 2 NEC, as opposed to 33.3% (2/6) in the control group. Deaths (*n* = 9) were due to respiratory reasons (two in each group), cardiac reasons (one in each group) and sepsis (two from control and one from the study group) [49]. While this study suggests a benefit to weight gain in LBW infants, the results on sepsis and NEC incidence are confounded by the more unfavorable baseline characteristics in the probiotic group.

*B. lactis* B94 exerted a significant protection against NEC in a RCT enrolling full-term infants with cyanotic congenital heart disease (CCHD) [50]. There were nine cases of culture-proven sepsis (18%) in the placebo group and two cases (4%) in the synbiotic group (*p* = 0.03). Length of NICU stay did not differ between the groups (26 (14–36) vs. 32 days (20–44), *p* = 0.07]. There were five cases of NEC (10%) in the placebo group and none in the synbiotic group (*p* = 0.03). The incidence of death was lower in synbiotic group (5 [10%] of 50 vs. 14 [28.0%] of 50, respectively; *p* = 0.04); however, the different distribution of the various cardiac diagnoses between groups poses a challenge to the definitive interpretation of the effect of the probiotic on the mortality rate. Taken together, these studies support a beneficial effect of *B. lactis* B94 in the prevention and progression of NEC, and perhaps mortality, as well as safety of this strain in newborns at risk of NEC.

### 2.2. Diarrhea in Acute Gastroenteritis

Two RCTs assessed the effect of *B. lactis* B94 on diarrhea outcomes in children with acute gastroenteritis. One study assessed the effect of the B94 + inulin synbiotic as an adjuvant to the standard treatments provided in the control group (i.e., oral rehydration therapy and rapid refeeding with a normal diet; n = 25/group). Enrolled children (5 mo–5 y old) were diagnosed with rotavirus-induced gastroenteritis using a latex agglutination stool test. Participants were rehydrated in the hospital, then follow-up was conducted by phone after discharge for number of diarrhea episodes, stool consistency, and vomiting. Diarrhea resolved significantly faster, by approximately 3 days, in the group receiving the probiotic (4.1 ± 1.3 days) compared to the controls (7.0 ± 1.6; *p* < 0.001). However, although the disease severity markers at baseline (dehydration score, hospitalization rate) were not different between groups, it cannot be excluded that differences in rotavirus vaccination rate between the probiotics and control groups could have positively influenced the outcome (16% vs. 4%) [51]. The second study assessed the effect of 5 × 10^10^ CFU of B94 + 900 mg of inulin (daily, for 5 days) on acute infectious diarrhea in children aged 2 mo–5 years [52]. On average, the synbiotic reduced the duration of diarrhea by 31 h compared to the placebo (3.9 ± 1.2 vs. 5.2 ± 1.3 days; *p* < 0.001), which resulted in a significantly lower number of diarrhea-type stools on the second (6.6 ± 3.4 vs. 9.3 ± 3.9; *p* < 0.001) and third days (5.5 ± 2.9 vs. 8.3 ± 3.01; *p* < 0.001). On the fifth day, there was significantly fewer diarrhea cases in the synbiotic group vs. placebo (38.9% vs. 17.7%; *p* = 0.002). Diagnosed pathogens were represented equally between groups (probiotic vs. placebo): unspecified (49.3% vs. 48%), rotavirus (33.7% vs. 36%), adenovirus (12.9% vs. 10%), *Salmonella* spp. (2.5% vs. 2.6%), and *Entamoeba histolytica* (2.5% vs. 2.6%). The reduction of diarrhea duration was more pronounced in the rotavirus-diagnosed subgroup (3.2 ± 1.3 vs. 6.6 ± 1.4 days, *p* < 0.001) and was not significant in adenovirus-infected infants. Diarrhea duration was also significantly shorter when the synbiotic was started within the first 24 h from onset compared to those who had started the synbiotic later (3.9 ± 1.1 vs. 4.8 ± 1.8 days; *p* = 0.002).

### 2.3. GI symptoms in Children with IBS

A prospective RCT was conducted to compare the efficacy of *B. lactis* B94 (n = 24; 5 × 10^9^ CFU, BID), the synbiotic B94 + inulin (n = 23; 5 × 10^9^ CFU + 900 mg inulin, BID), and inulin (n = 24; 900 mg, BID) in children aged between 4 and 16 years diagnosed with IBS according to Rome III [53]. There was no significant difference in the occurrence of symptoms or subtype distribution between groups at baseline. A sudden urge to defecate (66.2%), bloating after meals (64.8%), belching (64.8%), difficulty with defecation (63.3%), and a feeling of incomplete evacuation (56.3%) were the most frequently reported symptoms in this population, the least frequent being mucoid defecations (42.3%) and abdominal pain (daily in 19%, once a week in 34%, at least 3 days per month in 39%). Upon reassessment of symptoms at the end of the study (4 weeks), a significant improvement in bloating after meals, belching, and difficulty with defecation was observed in both groups who received B94, while none of the initially reported symptoms improved in the group receiving only inulin. Mucus in stools improved only in the synbiotics group. Full recovery (primary outcome defined as the significant improvement of all the initially reported symptoms) was observed in nine and seven participants (39.1% and 29.2%) in the synbiotic and probiotic groups, respectively, compared to three participants (12.5%) in the prebiotic group; this difference versus prebiotic alone was significant only for the synbiotics group.

### 2.4. Helicobacter Pylori Eradication and AAD

Three studies assessed the effect of B94 + inulin in 4–18-year-old children treated for *H. pylori* infections with standard triple antibiotic therapy [54,55,56]. In all studies, *H. pylori* infections were diagnosed by gastric biopsy (rapid urease test and/or histological examination) [54,55,56] or by fecal antigen positivity in some cases [54]. The standard triple therapy (STT) used the same antibiotics in all studies, namely a combination of amoxicillin (50 mg/kg/dose) and clarithromycin (15 mg/kg/dose), combined with 1 mg/kg/dose of either lansoprazole [54,55] or omeprazole [56] as the proton-pump inhibitor. Only the STT and probiotic administration regimens differed slightly between studies. Eradication was assessed between 4–8 weeks after the last STT intake using ^14^C-urea breath test [56] or fecal *H. pylori* antigen detection [54,55].

One study reported a significant increase in *H. pylori* eradication [55], while the two other studies reported a trend for an increased eradication rate in the synbiotic groups [54,56]. When considered together (n = 262 children), these studies suggest that *B. lactis* B94 could increase the likelihood of eradication treatment success by 23%, on average, when used as an adjuvant to standard triple eradication therapy (pooled RR = 1.23; 95%CI: 1.07–1.42; Z = 2.86; *p* < 0.0042). While the effect of B94 on eradication efficacy may require confirmation in a larger trial, the reduction in STT side effects by this probiotic was significant in all studies; *B. lactis* B94 significantly reduced diarrhea and abdominal pain, and tended to reduce dyspeptic symptoms [54,55,56].

## 3. Discussion and Perspectives

*B. lactis* strains are widely used in a variety of dairy products or fermented foods [57,58], and their use as probiotics has been extensively documented in newborns, children, and adults [26,59,60,61,62,63]. The *B. lactis* subspecies was described as a strict monophyletic bifidobacterial taxon [64]. Some of the beneficial effects are likely to be shared between strains. However, this cannot be extrapolated systematically since differences of potentially functional relevance were reported between some *B. lactis* strains despite the very small number of SNPs and INDELs in their whole genome sequences [40,65].

**Figure 1:**
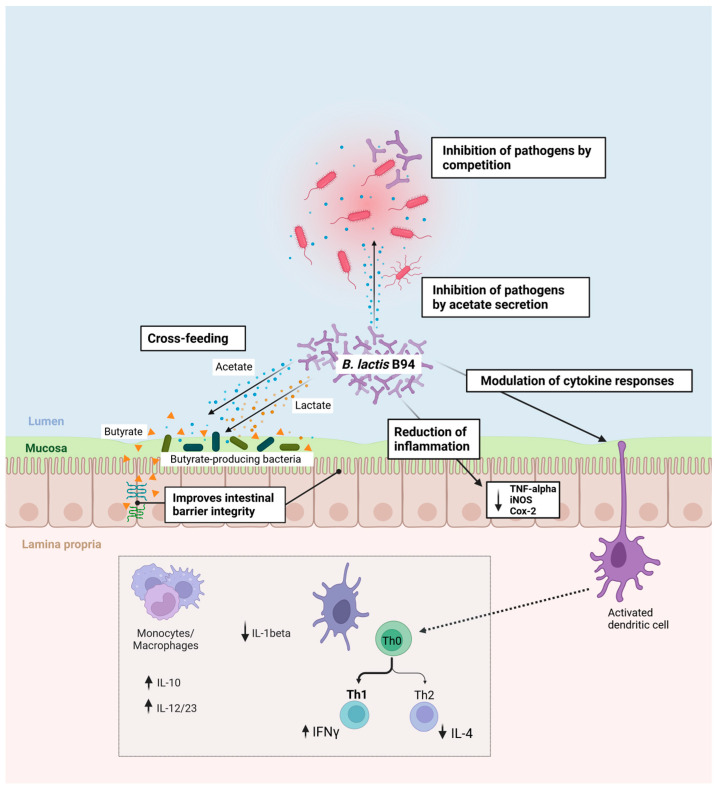
Summary of the potential modes of action behind the benefits of *B. lactis* B94 on gut health in children. *B. lactis* B94 may inhibit pathogens directly by competition or by secreting acetate. The acetate secreted by B94, with lactate as a co-substrate, can increase the production of butyrate by a cross-feeding mechanism with various butyrate-producing communities [69]. In turn, butyrate exerts a positive impact on the host, notably by contributing to the maintenance of an intact intestinal lining and barrier function. B94 was associated with a reduction in the levels of inflammation markers (TNF-α, iNOS, Cox-2) in the colon wall in a rodent model of colitis. B94 can also modulate cytokine responses; B94 favored a Th1 response over Th2 (increasing IFN-γ and reducing IL-4) and can stimulate IL-10 (and IL-12/23p40 to a lesser extent) while reducing IL-1β, as shown in cultured human peripheral blood cells. Created with BioRender.com (accessed on 19 September 2023).

Nevertheless, one indisputably shared characteristic among all *B. lactis* strains is their relative incapacity to grow on most HMOs compared to *B. infantis* due to the absence of nearly all HMO internalization and utilization genes from their genomes [66,67]. Of note, other *Bifidobacteria* strains with relevance to pediatric microbiota composition, such as *B. breve* and *B. bifidum*, are also devoid of multiple known HMO metabolism genes and, consequently, also displayed a relatively poor growth rate on HMOs in vitro compared to *B. infantis* [66,67]. Interestingly, *B. lactis* strains (as well as *B. adolescentis* and *B. catenulatum*) appear to possess the ability to grow weakly on 3-FL [66], including B94 (unpublished observations), which would require further investigation due to the complexity of HMO uptake and utilization mechanisms in *Bifidobacteria* and the possible existence of yet uncharacterized HMO utilization genes [67,68]. Despite increasing interest from the scientific community, research on HMOs and probiotic–HMO combinations is still in its infancy. While the concept of HMO utilization has emerged as a sought-after beneficial feature of “infant-type” probiotics, strains that generally do not consume HMOs should not be considered de facto as being of inferior value for newborns and pediatric populations, especially when their benefits are supported by clinical evidence and in vivo mechanistic insights (Figure 1).

Indeed, ideal HMO–probiotic pairing does not necessarily entail that the probiotic should consume its paired HMO. For instance, lower DSLNT levels in breast milk was identified as a predictor of NEC incidence, suggesting that, as shown in vivo, this HMO confers protection by a yet uncharacterized mechanism [70,71]; DSLNT could very well act via a direct immunomodulatory effect on host cells rather than through a microbiota-derived mechanism [72]. This possibility has raised the hypothesis that, should the NEC protection effect of DSNLT be host-mediated, a probiotic aimed at preventing NEC would preferably not consume this specific HMO and leave it available to exert its effects in situ [72]. *B. lactis* B94 protected against NEC in a multicenter trial including 400 infants, of whom the majority did receive breast milk (~77–79.5% with exclusive breast milk or mixed feeding vs. ~20.5–23% with exclusive formula feeding) [48]. While the beneficial effects of *B. lactis* B94 on the intestinal morphology and function, or its anti-inflammatory effects, are likely at play in its protective role against NEC, a potential synergistic effect with specific HMOs (such as DSLNT), although speculative at this point, could merit further attention.

In addition, *B. lactis* B94 could exert some beneficial effect on gut health by increasing acetate levels [21,38]. However, current knowledge on how SCFAs affect GI health in different populations (age and health/disease profiles) remains volatile; most conclusions in clinical settings are drawn by associations between health outcomes and fecal acetate levels used as a proxy but since SCFAs are also absorbed in the intestines, such correlations must be interpreted with caution. In healthy infants and newborns, fecal SCFA profiles appear to be influenced by the feeding mode. A recent meta-analysis showed that, even though total levels of SCFAs were higher in healthy formula-fed infants, the relative fecal acetate levels were lower than in their breastfed counterparts during the first month of life [73]. This may be related to the higher diversity and richness of the microbiota in formula-fed infants compared to breastfed in the first month, although the significant heterogeneity among the included studies prevented the authors from correlating SCFA levels with specific microbiota composition [73]. More research is required to characterize the contribution of acetate production by *B. lactis* B94 in its documented health benefits.

## 4. Conclusions

More studies are required to better define the mechanisms of action of *B. lactis* B94 in relation with various perinatal factors on microbiota composition, such as diet and disease, to better understand how this strain confers protection against NEC progression or diarrhea of various etiologies. Based on in vivo studies, the capacity of this strain to support the morphological integrity and function of the intestinal lining, to regulate immune responses in a model of allergic disease and reduce inflammation-induced mucosal damage, to increase acetate levels in stools, and to compete with intestinal pathogens may all contribute to the GI health benefits provided by *B. lactis* B94 in pediatric populations. Interesting avenues for research are the potential pairing with specific HMOs in breast milk and the potential benefits of these pairs on the incidence or progression of pediatric diseases.

## Figures and Tables

**Table 1:** Safety and preclinical studies on *B. lactis* B94.

Category	Results	References
Strain characterization and safety	Strain number CBS-118529 deposited at the *Centraalbureau voor Schimmelcultures* (Netherlands). Identity as part of the *B. lactis* monophyletic taxon was confirmed by WGS. No adverse effects were observed in toxicological analyses in mice fed with 1000 g/kg of *B. lactis* B94 for 13 weeks. No antibiotic resistance gene of clinical concern per EFSA guidelines. Presence of *Tet(W)* gene (all *B. lactis* strains) conferring moderate resistance to tetracycline with negligible potential for transfer. Sensitive to all other EFSA-required antibiotics in microdilutions assays (i.e., ampicillin, chloramphenicol, clindamycin, erythromycin, gentamycin, streptomycin, and vancomycin). Mutation of the rpoB (RNA polymerase β-subunit) housekeeping gene (in all publicly available *B. lactis* sequences; mutation also described in *B. longum* and *B. infantis* strains) conferring resistance to the anti-tubercular drug rifampicin.^1^ The general contraindications for probiotics apply to B94, including exerting caution or refraining from using in critically ill and immunosuppressed individuals, in individuals with a central catheter in place, or in children with short bowel syndrome (NNHPD; NPN 80077056, approving its use in newborns, infants, and adults). In the USA, the use of *B. lactis* B94 has been Generally Recognized as Safe (GRAS) by the Food and Drug Administration (FDA) in the form of a no question letter for addition to non-exempt milk-based term infant formula, issued in 2019.	[22,23,24,25,26,27]
*B. lactis* species safety	Included in several lists and inventories from authoritative bodies worldwide for safe use in foods, including the European Food Safety Authority (EFSA), the Australian Therapeutic Goods Administration (TGA), the Medicines Control Council in South Africa, and the Food Safety and Standards Authority of India, as well as in a positive list of strains to be used in functional foods/health foods in Korea and China.	[28,29,30,31,32,33]
Gastrointestinal tract (GIT) survival and adhesion to intestinal cells	*B. lactis* B94 survives in GIT-simulated conditions typically encountered at mealtime (pH 3–6), although being more susceptible to the very low pH encountered on an empty stomach (typically pH 1–2). Similar growth rate on agar after in vitro GIT-simulation. Viable *B. lactis* B94 (1.8 × 10^9^ CFU/g wet feces) was recovered in mice and human after oral intake. Adheres to intestinal epithelial cells in vitro.	[34,35,36]
Synergy with prebiotics	Several prebiotics were able to support the growth of *B. lactis* B94 in vitro when used as the sole carbon source, notably inulin, FOS, isomalto-oligosaccharides, xylo-oligosaccharides, and soybean oligosaccharides (SOS). The live strain was detected in the feces of mice with a standard diet for up to 3 days after gavage of a single bolus, and for up to 13 days in the presence of inulin and FOS.	[35,36].
Intestinal barrier integrity and function	Reduced inflammation-related intestinal damage and diarrhea in a rat TNBS-induced colitis model. Improved intestinal morphology in a rat model of D-galactosamine-induced liver failure, partially alleviated liver damage and gut microbiota dysbiosis, attenuated the elevation of MCP-1 levels, and normalized liver function parameters (AST, ALT, and bilirubin). B94 (with inulin) improved ileal histomorphology (increased villus height: crypt depth ratio), increased the total amount of short-chain fatty acids and the proportion of acetate, and significantly decreased the total aerobic bacteria and coliform counts in the caecum of broiler chickens. B94 (with inulin) promoted better nutrient utilization in broiler chickens, probably via its beneficial effects on intestinal morphology, which translated into an increased performance (growth and meat quality) and improved bone strength and mineral composition (Ca, P, Cu, Mn, Zn).	[37,38,39]
Acetate production	Produced high levels of acetate (up to ~20 g/L) under appropriate growth conditions and does not possess the acetate kinase gene mutation that was associated with a reduced acetate production capacity in another *B. lactis* strain. Increased the ratio of acetate among SCFAs in vivo (broiler chicken).	[38,40]
Immune defense and immunomodulatory effects	In vitro, *B. lactis* B94 exhibited a direct antilisterial effect in a plate assay and significantly reduced the growth of *E. coli* and *L. monocytogenes* by more than 2.5 log in contaminated salami batter fermented at 25 °C for 7 days when compared to levels found in salami batter fermented using a traditional meat starter. Mice pre-treated with B94 for 7 days before infection by *S. typhimurium* (single bolus challenge) displayed similar levels of *S. typhimurium* in their feces but did not develop symptoms of salmonellosis as opposed to those receiving pretreatment with another *B. lactis* strain or no probiotics. Reduced the damaging effect of inflammation on the intestinal lining in the rat TNBS-induced colitis model. ο Reduction in tumor necrosis factor (TNF)-α, inducible nitric oxide synthase (iNOS), and cyclooxygenase-2 (COX-2) levels in the colon wall. In cultured human peripheral blood-derived monocytes (PBMCs) from 10 healthy donors, B94 stimulated the production of high levels of IL-10 and, to a lesser extent, IL-12. In cultured T-cells challenged with the antigen-independent T-cell activator Concanavalin A, B94 increased INF-γ and decreased IL-4, inversing the IFN-γ/IL-4 ratio and favoring a Th1 response over Th2. A polarized Th1 response was also observed in a murine allergy model; total IgE was suppressed, and neutrophilic infiltration was reduced in the airways of the mice. In a model of *H. pylori*-induced gastritis, B94 reduced diarrhea, gastric neutrophil infiltration, and IL-1β levels, and increased IL-10 levels. The B94-treated mice displayed a significant increase in IL-12/23p40, indicating a Th1 immune response, with a reduction in circulating *H. pylori* IgG consistent with an improvement in the mucosal barrier integrity (similar *H. pylori* levels in feces).	[37,41,42,43,44,45]

^1^ A similar intrinsic resistance to rifampicin in *B. longum* W11 was shown to provide additional health benefits following concomitant use with rifampicin in patients with symptomatic uncomplicated diverticular disease [27].
